# Supplementary material for: Natural variation of BSK3 tunes brassinosteroid signaling to regulate root foraging under low nitrogen
Source: Nat Commun. 2019 May 30;10:2378. doi: 10.1038/s41467-019-10331-9 (PMC6542857; doi:10.1038/s41467-019-10331-9)
Supplement: Supplementary file 1 — Supplementary Information [file 41467_2019_10331_MOESM1_ESM.pdf]

Supplementary Information

Natural variation of BSK3 tunes brassinosteroid signaling to regulate root foraging under low nitrogen

Jia et al.

* Correspondence: Nicolaus von Wirén

E-mail: vonwiren@ipk-gatersleben.de

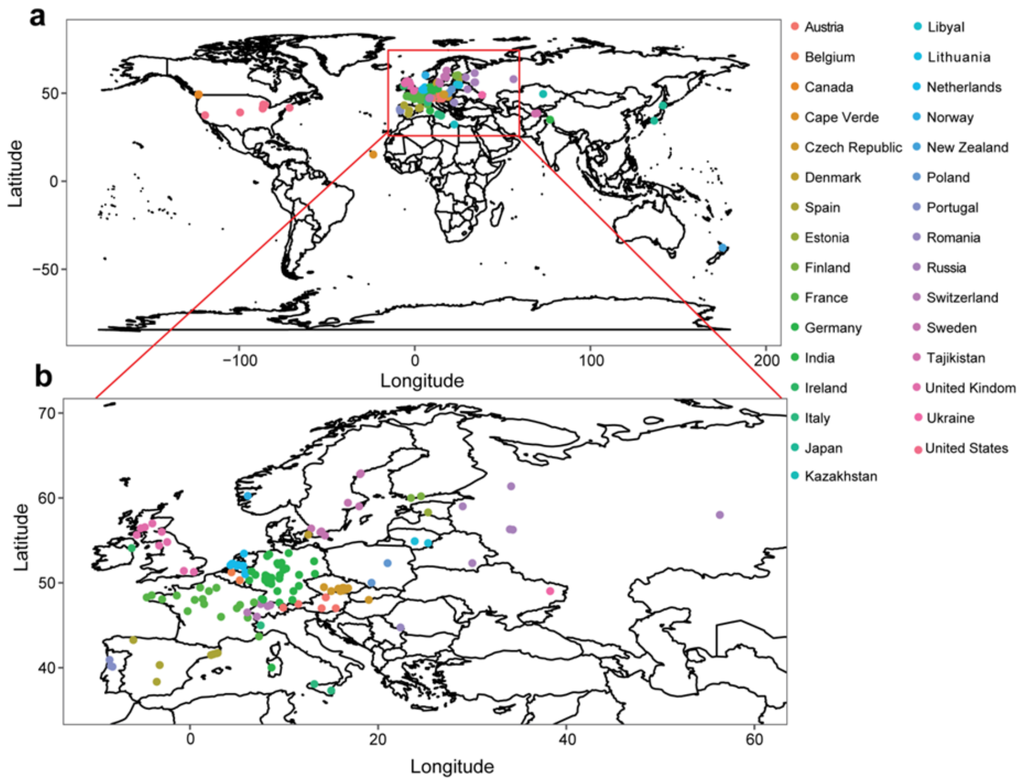

Supplementary Figure 1. Geographic origin of the 200 accession lines used in the present study. (a) The global map of 200 lines originating from 31 countries. **(b)** A zoomed-in regional map for accessions contained within the area (-10˚ to 60˚ E and 35˚ to 70˚ N) highlighted by a red frame in **(a)**. Dots represent the sampling site and color indicates the country of origin. The distribution of accessions is visualized by R package “rworldmap”.

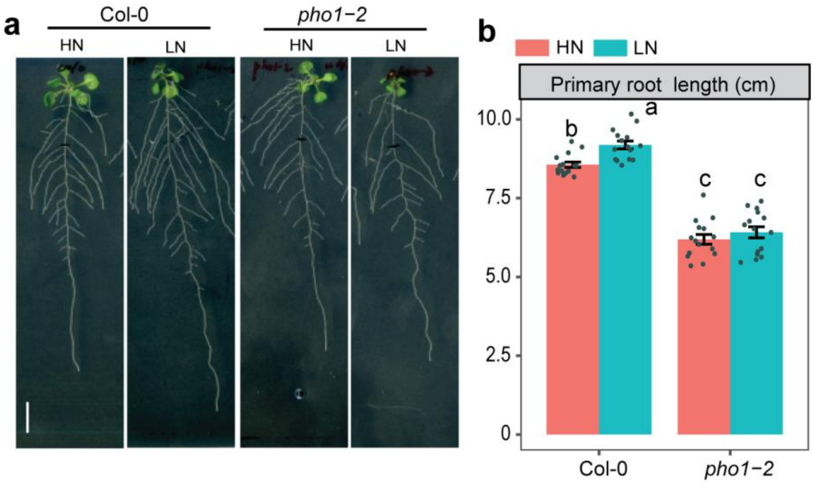

Supplementary Figure 2. Primary root length of the *pho1-2* mutant in response to low N. (a-b) Appearance of plants **(a)** and primary root length **(b)** of wild-type (Col-0) and *pho1-2* mutant plants grown at high N (HN, 11.4 mM N) or low N (LN, 0.55 mM N) conditions. Seven day-old seedlings pre-cultured on 11.4 mM N were then transferred to solid agar media containing either HN or LN. Lengths of primary root were measured after 9 days. Bars represent means ± s.e.m. (n = 15 independent biological replicates). Different letters indicate significant differences at P < 0.05 according to one-way ANOVA and post-hoc Tukey test. Scale bar, 1 cm.

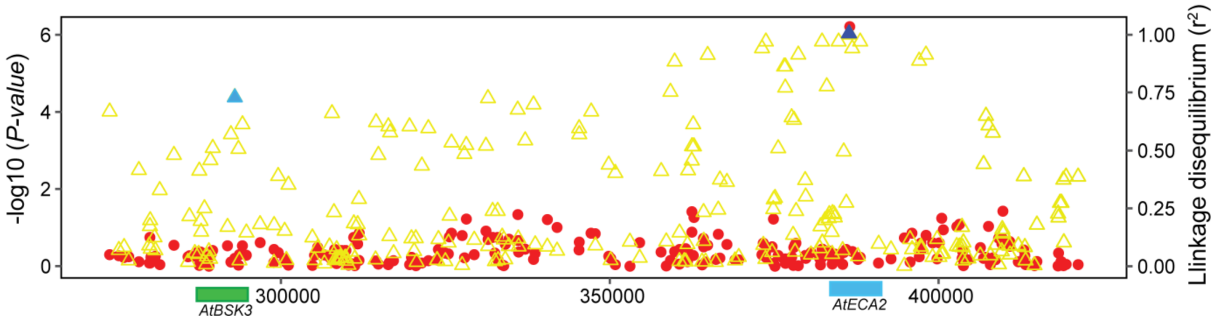

Supplementary Figure 3. Manhattan plot of a local association scan using MLMM and the linkage disequilibrium pattern in the region surrounding *BSK3*. Red dots represent $-\log_{10}$(*p-value*) for the association between each marker and primary root length at low N. Triangles indicate the r^2 value for the GWA peak (in dark blue) and surrounding SNPs. The SNP in the *BSK3* (G1353A) is marked in light blue. The boxes on the horizontal axis indicate the position of the *BSK3* (in green) and *AtECA2* (in light blue).

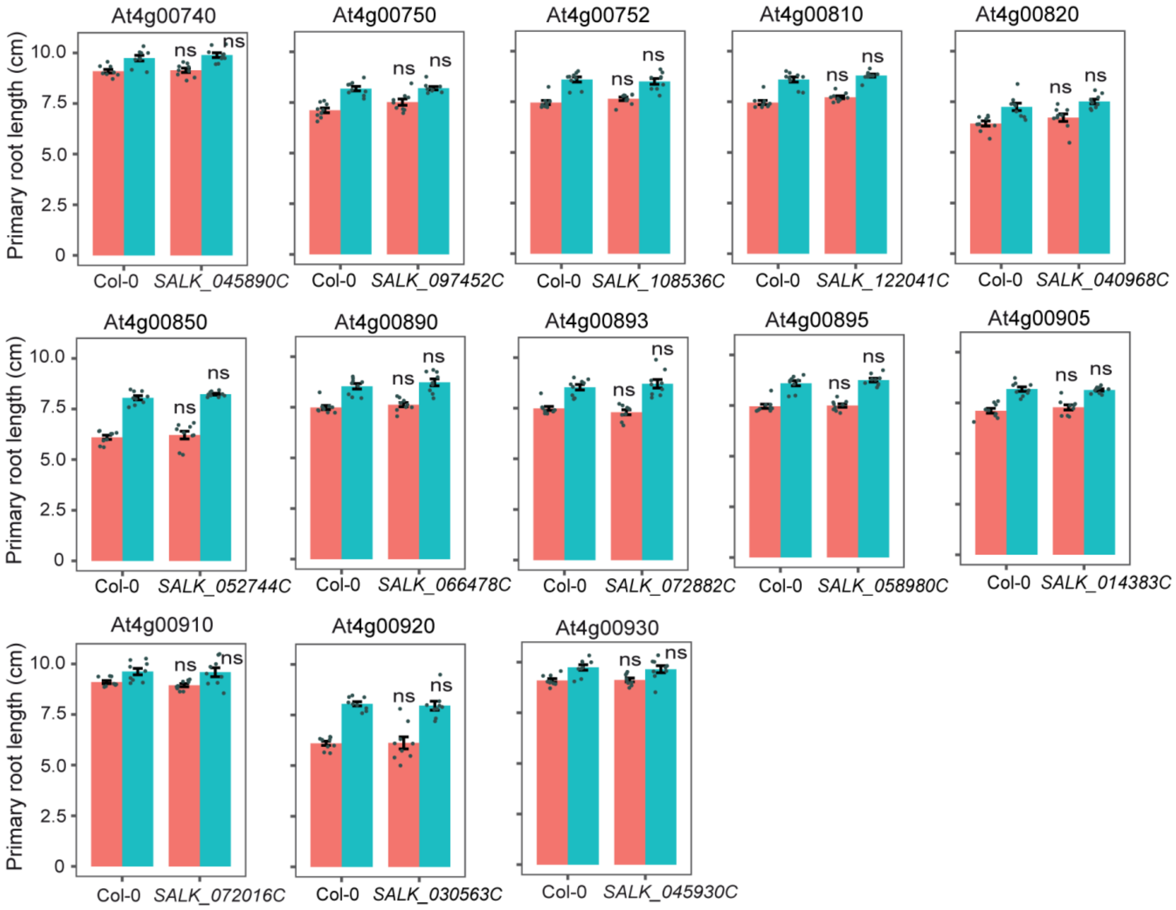

Supplementary Figure 4. Primary root length of T-DNA insertion lines of genes contained within the selected genomic interval. Seven day-old seedlings of wild-type and T-DNA insertion lines pre-cultured on 11.4 mM N were transferred to solid agar media containing either high N (HN, 11.4 mM N) or low N (LN, 0.55 mM N). Primary root length was determined after 9 days. Bars represent means ± s.e.m. (n = 9 independent biological replicates). ns denotes no significant difference to wild-type at respective N condition according to Welch's t-test ($P > 0.05$).

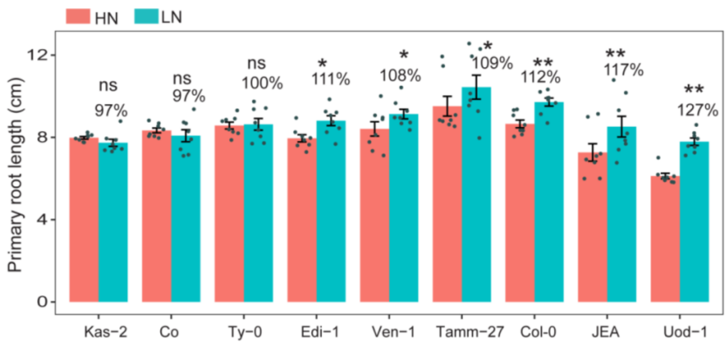

Supplementary Figure 5. Primary root length of nine natural accessions used for gene expression analysis. Accessions Kas-2, Co, Ty-0, Tamm-27, JEA and Uod-1 express the P-type BSK3 protein, and Edi-0, Ven-1 and Col-0 the L-type protein. Seven day-old seedlings pre-cultured on 11.4 mM N were transferred to solid agar containing either high N (HN, 11.4 mM N) or low N (LN, 0.55 mM N). Primary root length was determined after 9 days. Numbers above columns indicate percent changes under LN versus HN for primary root length. Bars represent means ± s.e.m. (n = 8 independent biological replicates). Asterisks indicate statistically significant differences between two N conditions for each genotype according to Welch's t-test (*P < 0.05; **P < 0.01; ns, not significant).

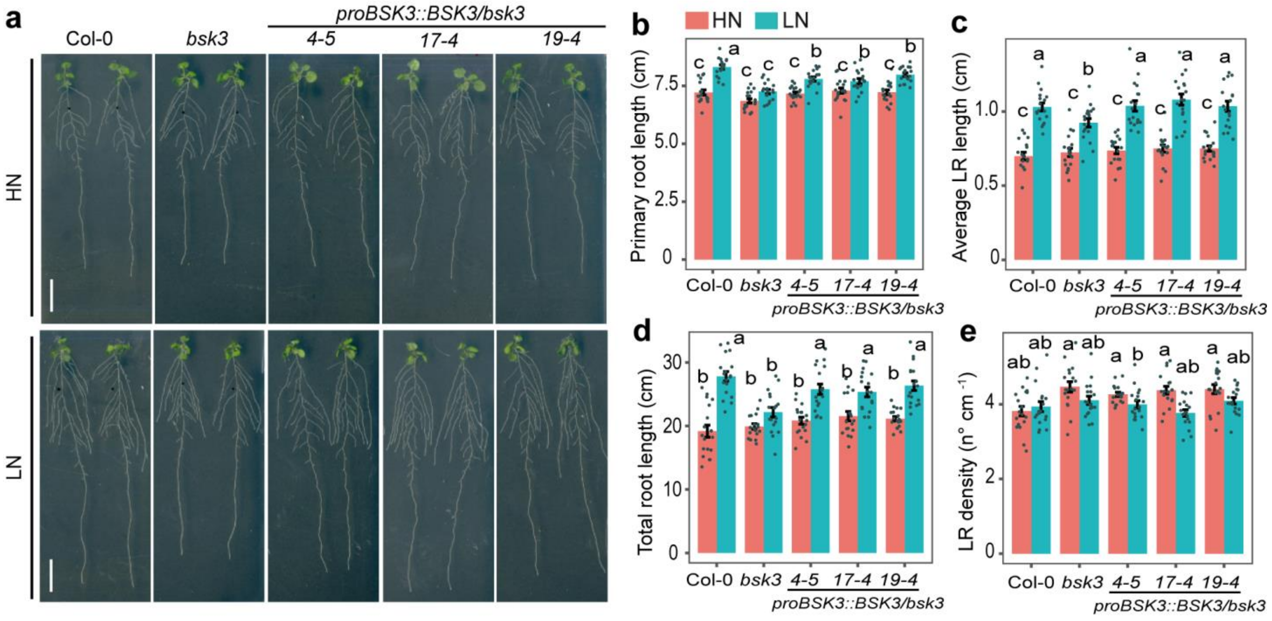

Supplementary Figure 6. Root architecture under differential N availability of the *bsk3* single mutant and of mutants complemented with genomic *BSK3*. Appearance of plants **(a)**, primary root length **(b)**, average lateral root length **(c)**, total root length **(d)** and lateral root density **(e)** of wild type (Col-0), *bsk3* and three independent T3 transformants complemented with genomic *BSK3* from Col-0. Seven day-old seedlings were pre-cultured on 11.4 mM N and then transferred to solid agar media containing either high N (HN, 11.4 mM N) or low N (LN, 0.55 mM N). Root system architecture was assessed after 9 days. Bars represent means ± s.e.m. (n = 18 independent biological replicates). Different letters indicate significant differences at P < 0.05 according to one-way ANOVA and post-hoc Tukey test. Scale bars, 1 cm.

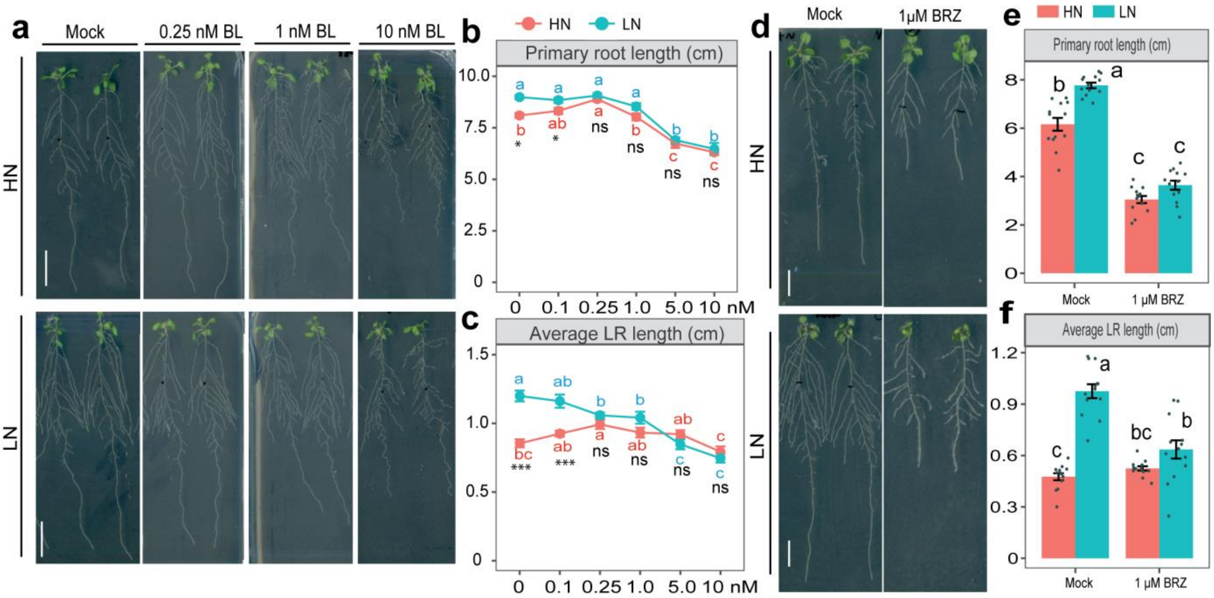

Supplementary Figure 7. Root growth of Col-0 in response to BL and BRZ under different N availabilities. Seven-day-old wild-type (Col-0) seedlings were pre-cultured on 11.4 mM N and then transferred to solid agar media containing either high N (HN, 11.4 mM N) or low N (LN, 0.55 mM N) in the presence or absence of indicated concentrations of 24-epibrassinolide (BL) or brassinazole (BRZ). Root system architecture was assessed after 9 days. **(a-c)** Appearance of plants **(a)**, primary root length **(b)** and average lateral root length **(c)** of Col-0 plants grown under BL supply**.** **(d-f)** Appearance of plants **(d),** primary root length **(e)** and average lateral root length **(f)** of Col-0 plants grown in absence or presence of BRZ. Bars represent means ± s.e.m. (*n* = 13 independent biological replicates). Different letters indicate significant differences at $P < 0.05$ according to one-way ANOVA and post-hoc Tukey test within respective N treatment (in **b** and **c**) or across N and BZR treatments (in **d** and **e**). Asterisks indicate statistically significant differences between two N treatments at individual BR concentration (in **b** and **c**) according to Welch's *t*-test (**P* < 0.05, ****P* < 0.001, ns, not significant). Scale bars, 1 cm.

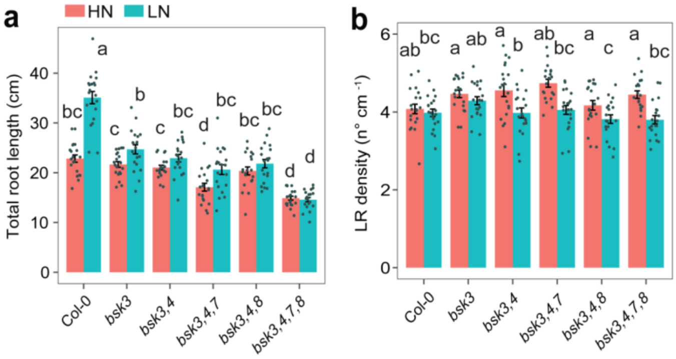

Supplementary Figure 8. Total root length and lateral root density of *bsk* mutants in response to low N availability. (a-b) Total root length **(a)** and lateral root density **(b)** of wild type (Col-0), *bsk3* as well as *bsk* multiple knockout mutants. Seven day-old seedlings pre-cultured on 11.4 mM N were then transferred to solid agar media containing either high N (HN, 11.4 mM N) or low N (LN, 0.55 mM N). Total root length and lateral root density were assessed after 9 days. Bars represent means ± s.e.m. (*n* = 20 independent biological replicates). Different letters indicate significant differences at $P < 0.05$ according to one-way ANOVA and post-hoc Tukey test.

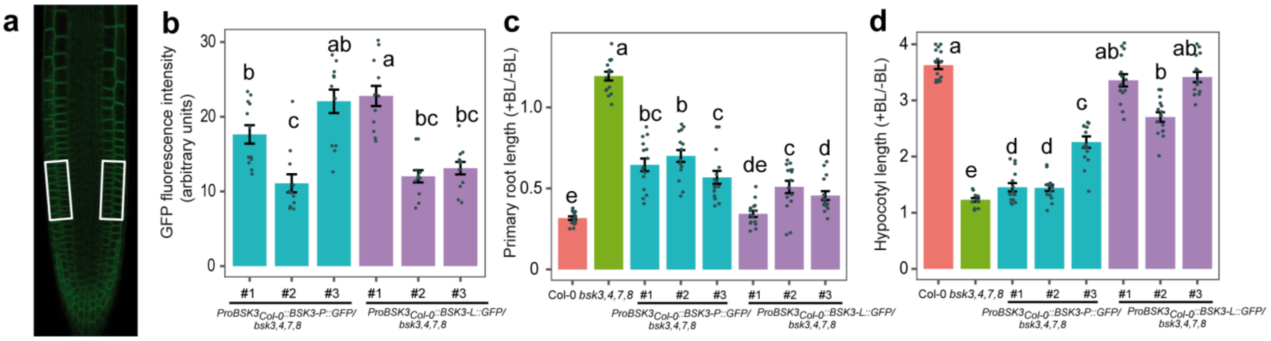

Supplementary Figure 9. BSK3-L-GFP confers higher BR sensitivity than BSK3-P-GFP. (a) Schematic representation of GFP signals quantification in roots. White frames indicate the areas in which GFP signals were quantified. Values of two measurements per root were averaged. **(b)** Protein levels of BSK3 as indicated by the GFP fluorescence intensity in three independent transgenic lines expressing *proBSK3*$_{Col\text{-}0}$*::BSK3-L::GFP* or *proBSK3*$_{Col\text{-}0}$*::BSK3-P::GFP* in *bsk3,4,7,8* background. Bars represent means ± s.e.m. (n = 12 independent biological replicates). **(c-d)** Primary root **(c)** and hypocotyl **(d)** growth response to exogenous application of 1 µM 24-epibrassinolide (+BL) or the equivalent amount of ethanol (-BL) for Col-0, *bsk3,4,7,8* and independent transgenic lines carrying BSK3-P-GFP or BSK3-L-GFP. Bars represent means ± s.e.m. (n = 15 independent biological replicates). Different letters indicate significant differences at $P < 0.05$ according to one-way ANOVA and post-hoc Tukey test.

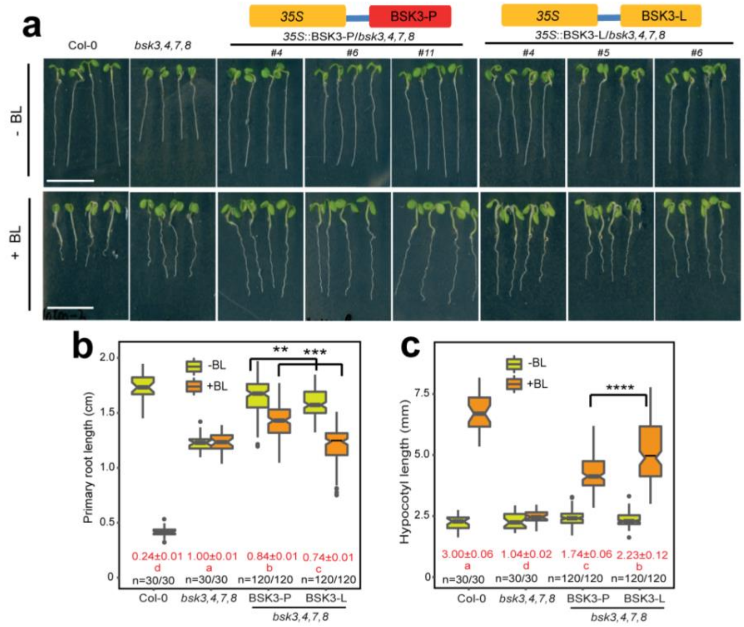

Supplementary Figure 10. Overexpression of BSK3-L confers higher BR sensitivity than BSK3-P. (a-c) Brassinosteroid sensitivity of wild type (Col-0), *bsk3,4,7,8* and independent transgenic lines expressing the sequences coding for either the BSK3-P or BSK3-L variant under control of the CaMV *35S* promoter. **(a-c)** Appearance of 6 day-old plants **(a)**, primary root length **(b)** and hypocotyl length grown under +BL and –BL. **(c)**. For each construct, 12 independent transgenic T2 lines were germinated on solid half-strength MS agar media containing 1 µM 24-epibrassinolide (+BL) or the equivalent amount of ethanol (-BL). Primary root length and hypocotyl length were assessed 6 days after germination. Horizontal lines show medians; box limits indicate the 25th and 75th percentiles; whiskers extend to 5th and 95th percentiles. Asterisks indicate statistically significant differences between two BSK3 protein haplotypes according to Welch's *t*-test (**$P < 0.001$; ***$P < 0.001$; ****$P < 0.001$). Numbers in red indicate relative root or hypocotyl length (+BL/-BL). Different letters indicate significant differences at $P < 0.05$ according to one-way ANOVA and post-hoc Tukey test. Scale bars, 1 cm.

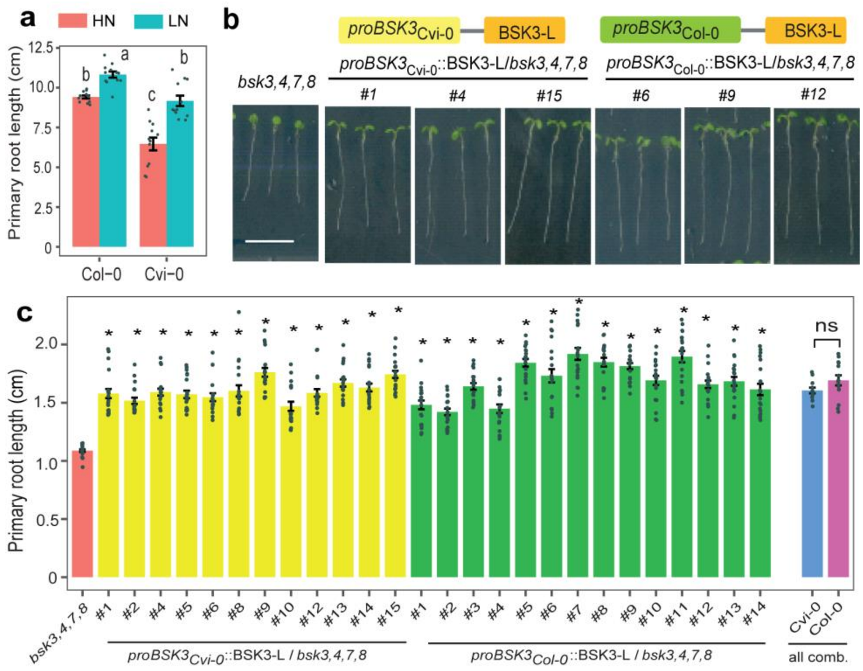

Supplementary Figure 11. Natural variation of root length is not associated with *BSK3* promoter variants. (a) Primary roots of Col-0 and Cvi-0 showing differential length and responsiveness to low N. Seven day-old seedlings pre-cultured on 11.4 mM N were transferred to solid agar containing either high N (HN, 11.4 mM N) or low N (LN, 0.55 mM N). Primary root length was determined after 9 days. Bars represent means ± s.e.m. (n = 12 independent biological replicates). Different letters indicate significant differences at $P < 0.05$ according to one-way ANOVA and post-hoc Tukey test. **(b)** Schematic of transgenic constructs used for complementation of the *bsk3,4,7,8* quadruple mutant and representative photographs of 6 day-old plants. Three representative lines for each construct are shown. Scale bar, 1 cm. **(c)** Primary root length of *bsk3,4,7,8* and 12 or 14 independent lines expressing BSK3-L under the control of *BSK3* promoter from either Cvi-0 or Col-0. Average values of 12 or 14 independent lines for each construct are also shown (all comb.) Primary root length was assessed 6 days after germination. The data for *proBSK3*$_{Col\text{-}0}$*::BSK3-L / bsk3,4,7,8* are also shown in Fig. 3a and b, since the experiment with the two *BSK3* promoter variants and the BSK3 protein types was carried out at the same time. Asterisks indicate statistically significant differences between complementation lines and *bsk3,4,7,8* mutant. There was no significant difference between two promoters according to Welch's t-test (*$P < 0.001$; ns, not significant).

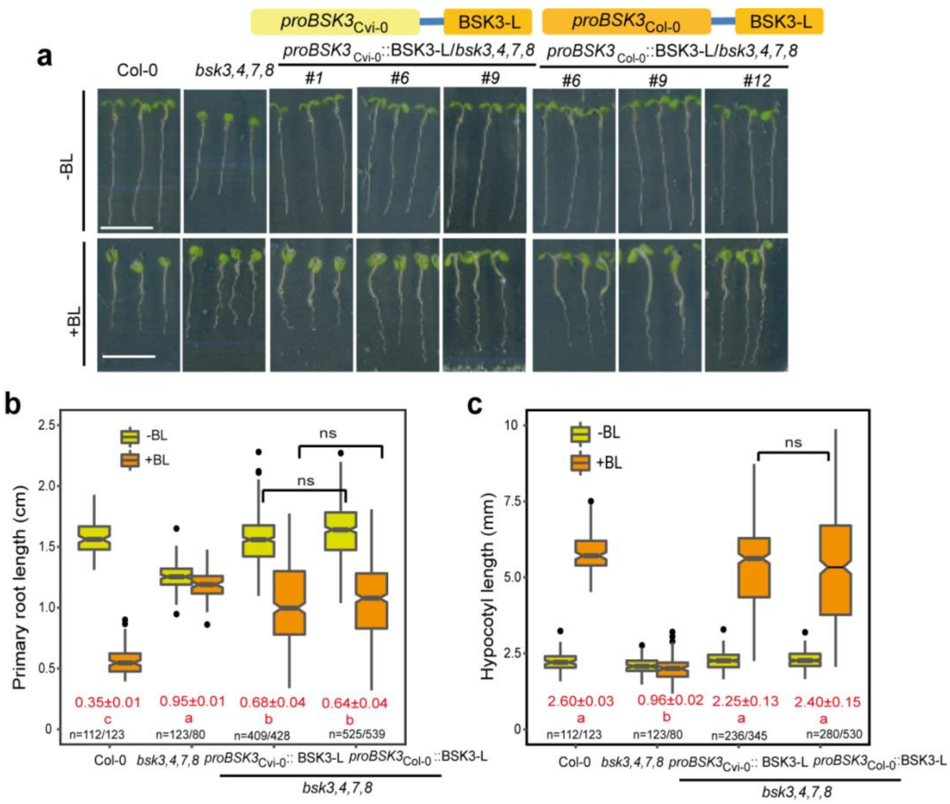

Supplementary Figure 12. Genotypic variation in the *BSK3* promoter sequence is not associated with brassinosteroid sensitivity. (a-c) Brassinosteroid sensitivity of wild type (Col-0), *bsk3,4,7,8* quadruple mutant and independent transgenic lines expressing the sequence coding for the BSK3-L protein variant under the control of either $BSK3_{Col\text{-}0}$ or $BSK3_{Cvi\text{-}0}$ promoter in *bsk3,4,7,8*. Appearance of plants **(a)**, primary root length **(b)** and hypocotyl length **(c)** 6 days after germination. For each construct, 12 and 14 independent transgenic T2 lines for $BSK3_{Cvi\text{-}0}$ or $BSK3_{Col\text{-}0}$ promoter were germinated on solid half-strength MS agar media containing 1 µM 24-epibrassinolide (+BL) or the equivalent amount of ethanol (-BL). Primary root length and hypocotyl length were assessed 6 days after germination. Horizontal lines show medians; box limits indicate the 25th and 75th percentiles; whiskers extend to 5th and 95th percentiles. The data for Col-0, *bsk3,4,7,8* and $proBSK3_{Col\text{-}0}$*::BSK3-L / bsk3,4,7,8* is also shown in Fig. 4b-c, since the experiment with the two *BSK3* promoter variants and the BSK3 protein types was carried out at the same time. There was no significant difference between two promoters according to Welch's *t*-test (ns, not significant). Scale bars, 1 cm. Numbers in red indicate relative root or hypocotyl length (+BL/-BL). Different letters indicate significant differences at $P < 0.05$ according to one-way ANOVA and post-hoc Tukey test.

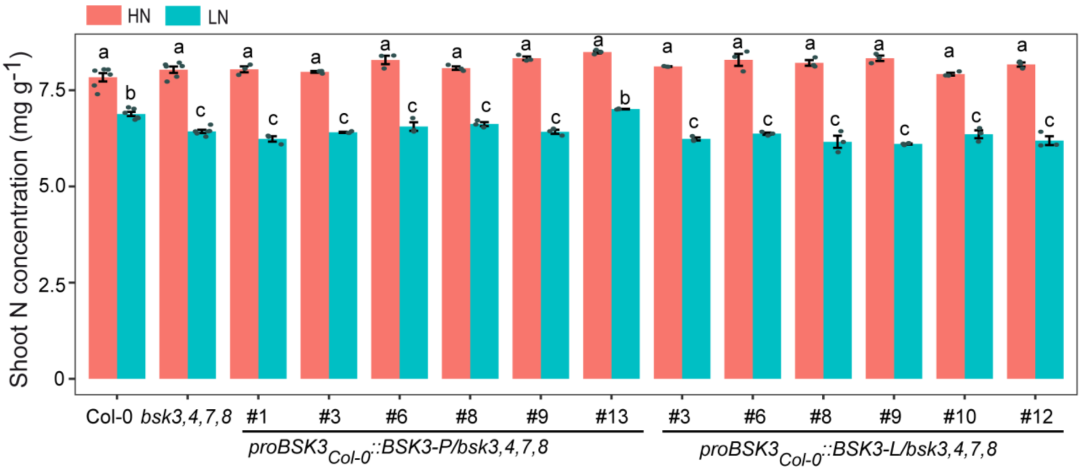

Supplementary Figure 13. Shoot N concentrations of plants grown under different N availabilities. Seven-day-old seedlings were pre-cultured on 11.4 mM N and then transferred to solid agar containing either high N (HN, 11.4 mM N) or low N (LN, 0.55 mM N). Shoots were collected for N concentration determination 9 days after transfer. Bars represent means ± s.e.m.(n = 6 independent biological replicates for Col-0 and *bsk3,4,7,8*, and 3 for individual transgenic allelic complementation lines). Different letters indicate significant differences at $P < 0.05$ according to one-way ANOVA and post-hoc Tukey test.

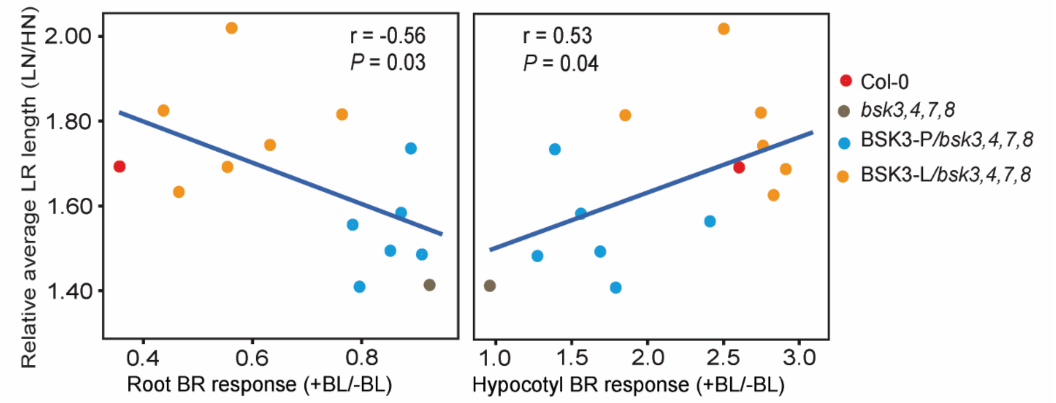

Supplementary Figure 14. Pearson correlation between lateral root responsiveness to N and BR-dependent root or hypocotyl elongation. The data used for the correlation analyses derived from experiments shown in Fig. 5b and Fig. 4b and c.

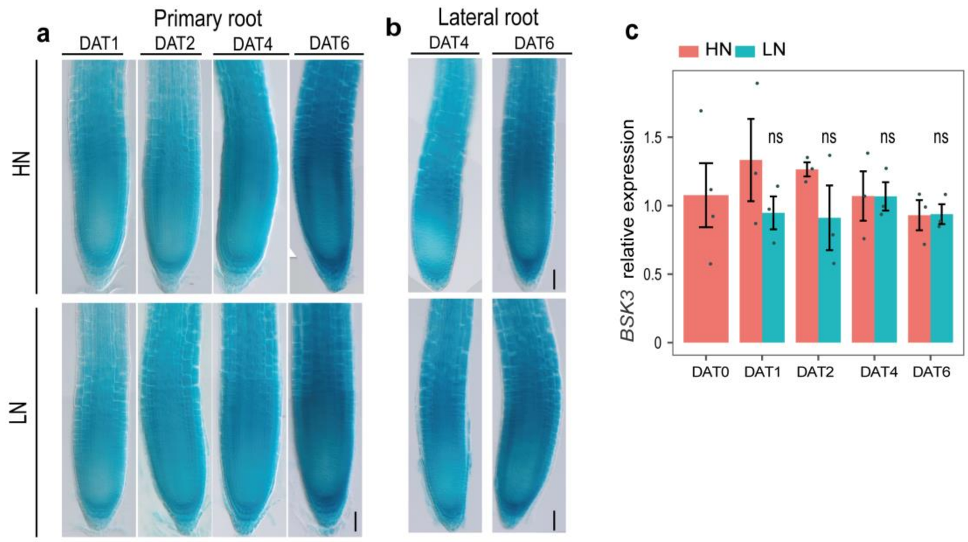

Supplementary Figure 15. Time course of *BSK3* expression in response to low N. *ProBSK3*-dependent GUS activity **(a**, **b)** and *BSK3* transcript levels **(c)** in response to N availability. (**a**-**b**) *ProBSK3*-dependent GUS activity was assessed in primary **(a)** and lateral root tips **(b)**. Seven day-old seedlings were pre-cultured on 11.4 mM N and then transferred to solid agar containing either high N (HN, 11.4 mM N) or low N (LN, 0.55 mM N). Samples for GUS activity staining and *qPCR* analysis were taken at the indicated day after transfer (DAT). Representative images (*n* =15) are shown. *BSK3* transcript levels were assessed in whole roots by *qPCR* analysis and normalized to *ACT2* and *UBQ10*. Bars represent means ±s.e.m. (*n* = 4 independent biological replicates for DAT0 and 3 for DAT1/2/4/6). No significant difference was detected between N treatments according to Welch's *t*-test (*P* > 0.05; ns, not significant). Scale bars, 100 µm.

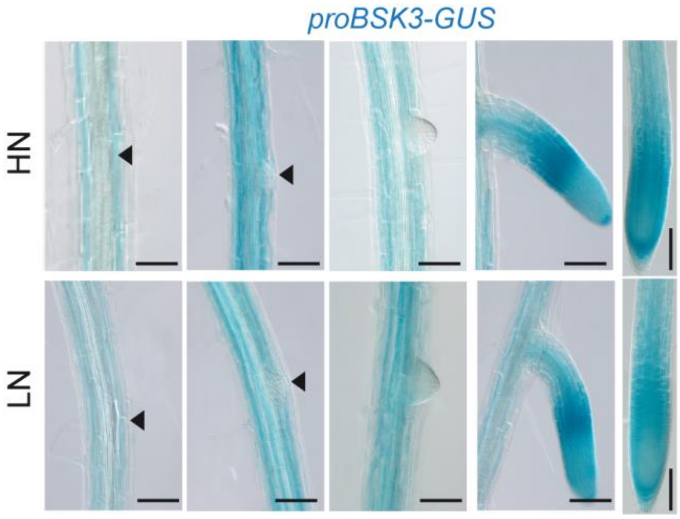

Supplementary Figure 16. *BSK3* promoter activity during lateral root development under different N availabilities. *ProBSK3*-dependent GUS activity was assessed in lateral root tips and during several stages of lateral root development**.** Arrowheads point to non-emerged lateral roots. Representative images (*n* =15) are shown. Scale bars, 100 µm.

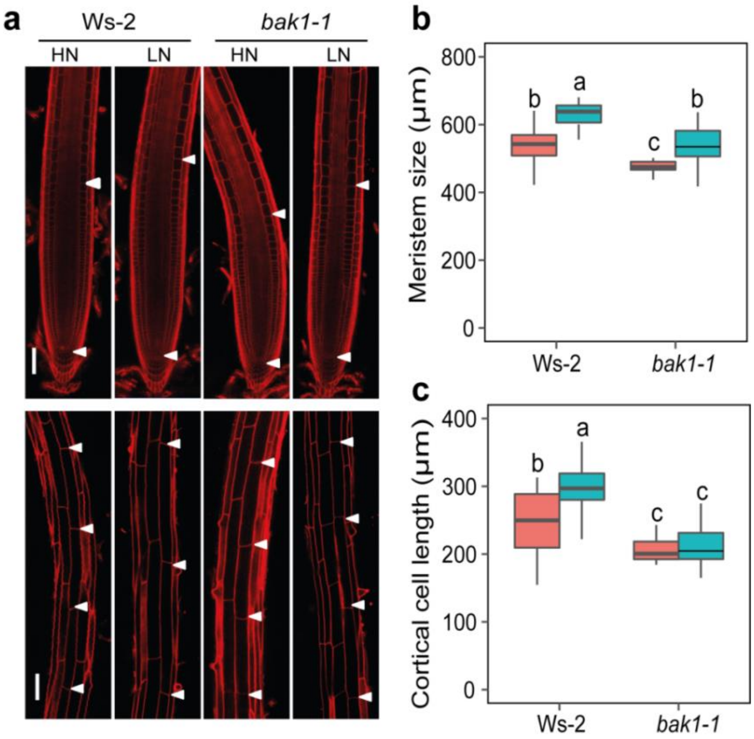

Supplementary Figure 17. Cellular traits of the primary root of *bak1-1* mutant under high or low N availability. Seven-day-old seedlings pre-cultured on 11.4 mM N were transferred to solid agar media containing either high N (HN, 11.4 mM N) or low N (LN, 0.55 mM N). Meristem size and cortical cell length were determined after 9 days. (**a**) Representative confocal images of root meristems (upper panel) and mature cortical cells (bottom panel) of wild-type (Ws-2) and *bak1-1* plants grown under HN or LN. In the upper panel, white arrowheads indicate the position of quiescent center (QC) and the boundary between the meristematic zone and elongation zone, while in the bottom panel they indicate the boundaries of two consecutive cortical cells. Scale bars, 100 µm. (**b**-**c**) Length of meristem **(b)** and mature cortical cells **(c)** of wild-type (Ws-2) and *bak1-1* (n = 15 independent biological replicates). Horizontal lines show medians; box limits indicate the 25th and 75th percentiles; whiskers extend to 5th and 95th percentiles. Different letters indicate significant differences at $P < 0.05$ according to one-way ANOVA and post-hoc Tukey test.

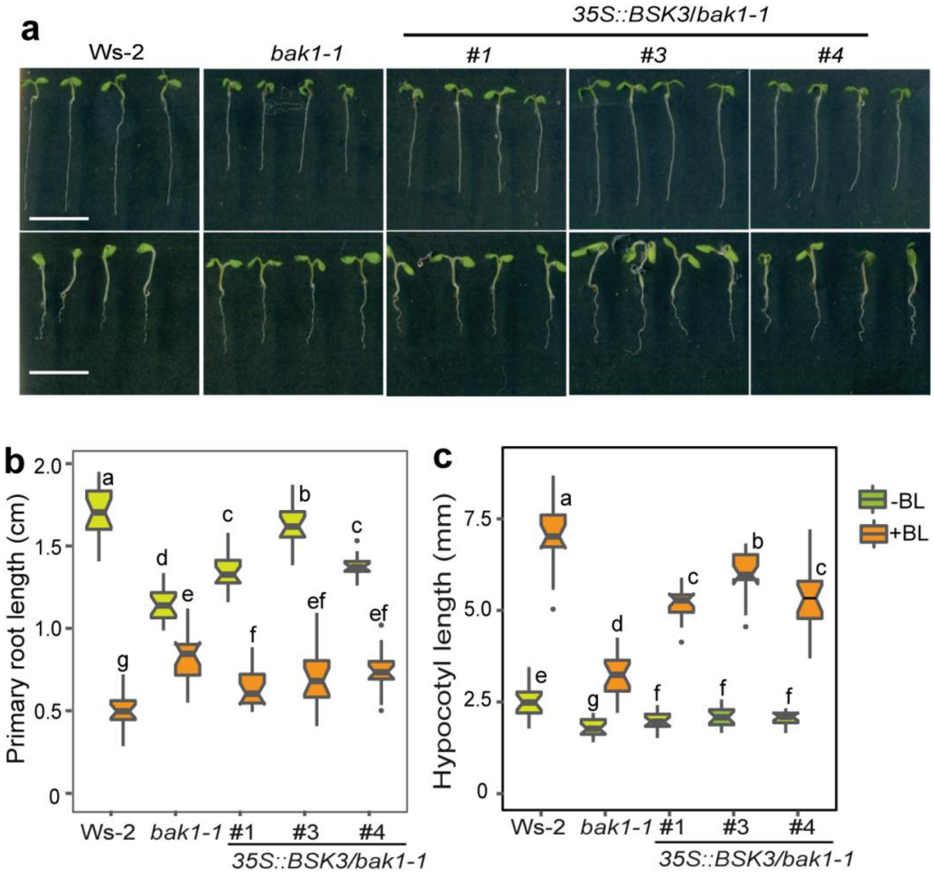

Supplementary Figure 18. Overexpression of *BSK3* increases BR sensitivity of the *bak1-1* mutant. (a-c) BR sensitivity of wild-type (Ws-2), *bak1-1* mutant and three independent T2 lines ectopically expressing *BSK3* in the *bak1-1* background under control of the *Ca MV35S* promoter. Appearance of plants **(a)**, primary root length **(b)** and hypocotyl length **(c)** 6 days after germination (*n* = 20 independent biological replicates). Seeds were germinated on solid half-strength MS agar media containing 1 µM 24-epibrassinolide (+BL) or the equivalent amount of ethanol (-BL). Primary root length and hypocotyl length were assessed 6 days after germination. Horizontal lines show medians; box limits indicate the 25th and 75th percentiles; whiskers extend to 5th and 95th percentiles. Different letters indicate significant differences at $P < 0.05$ according to one-way ANOVA and post-hoc Tukey test. Scale bars, 1 cm.

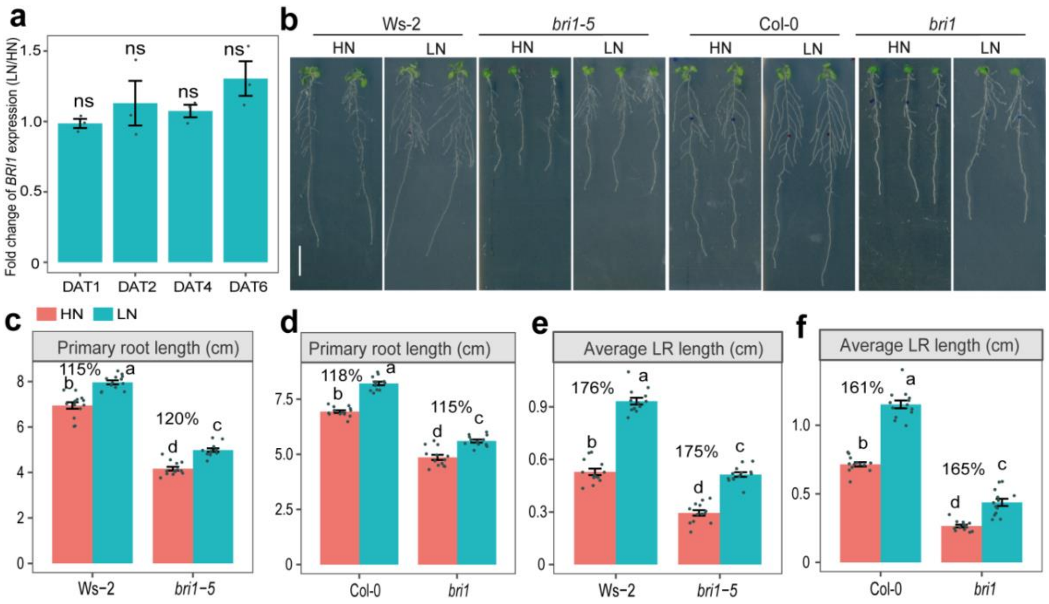

Supplementary Figure 19. Transcriptional response of *BRI1* to low N and phenotypic analysis of two *bri1* mutant plants. (a) Relative change of *BRI1* transcript levels in roots during growth on low N. Seven-day-old seedlings pre-cultured on 11.4 mM N were transferred to solid agar media containing either high N (HN, 11.4 mM N) or low N (LN, 0.55 mM N). *BRI1* transcript levels were assessed in whole roots by *q*PCR analysis and normalized to *ACT2* and *UBQ10*. Bars represent means ± s.e.m.(n = 3 independent biological replicates). DAT, day after transfer. No significant difference between two N treatments at each time points was detected according to Welch's *t*-test (P > 0.05; ns, not significant). **(b-f)** Appearance of plants **(b),** primary root length **(c-d)** and average lateral root length **(e-f)** of wild-type (Col-0 and Ws-2) and *bri1* mutant plants grown under high N (HN) or low N (LN) condition. Seven day-old seedlings pre-cultured on 11.4 mM N were transferred to solid agar containing either HN or LN. Root system architecture was assessed after 9 days. Bars represent means ± s.e.m.(n = 13 independent biological replicates). Different letters indicate significant differences at P < 0.05 according to one-way ANOVA and post-hoc Tukey test. Scale bar, 1 cm. Numbers above columns indicate percent changes under LN versus HN for primary and average lateral root length. No significant differences were detected between mutants and wild type according to Welch's *t*-test (P > 0.05).

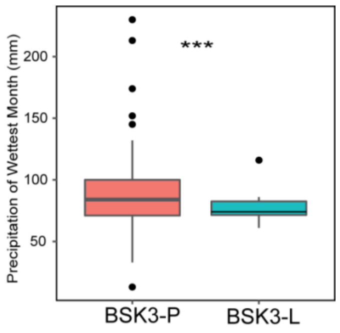

Supplementary Figure 20. Allelic variants of BSK3 associate with precipitation. Boxplots showing the association between BSK3-P or BSK3-L protein-coding haplotypes and precipitation during the wettest month in the geographic location from where the accession lines were collected (n = 89 and 26 accessions for P and L haplotypes, respectively). Horizontal lines show medians; box limits indicate the 25th and 75th percentiles; whiskers extend to 5th and 95th percentiles. Asterisks indicate statistically significant differences between two BSK3 protein haplotypes according to Welch's *t*-test (***P < 0.001).

Supplementary Table 1. Single nucleotide polymorphism (SNP) identified in the coding sequences (CDS) of 139 natural accessions.

Polymorphisms	Position in the CDS[a]	Allele count[b]	Mutation	MAF>5%
T/G	186	138/1	synonymous	no
G/A	330	138/1	synonymous	no
C/A	403	138/1	non-synonymous, P to T	no
C/T	415	137/2	synonymous	no
T/C	777	138/1	synonymous	no
A/C	816	136/3	synonymous	no
C/T	956	105/34	non-synonymous, P to L	yes
C/T	1203	138/1	synonymous	no
G/A	1353	110/29	synonymous	yes
T/A	1377	138/1	synonymous	no
A/G	1413	131/8	synonymous	yes

a) Position of SNP in the coding region relative to the start codon ATG without consideration of intron.
b) The number of accessions carrying each allele in 139 re-sequenced accessions.

Supplementary Table 2. Correlation of primary root length and climate variables. Pearson correlation between primary root length of 115 accessions grown on agar plates with high N (HN, 11.4 mM N) or low N (LN, 0.55 mM N) and climate variables (19 climate scenarios, latitude and longitude) associated with the original sampling sites of these accessions in their natural habitats. Only one significant correlation coefficient ($P < 0.05$) is found. Non-significant correlations are denoted by "ns".

	Primary root length	
Climate variables	HN	LN
Annual Mean Temperature	ns	ns
Mean Diurnal Range	ns	ns
Isothermality	ns	ns
Temperature Seasonality	ns	ns
Max Temperature of Warmest Month	ns	ns
Min Temperature of Coldest Month	ns	ns
Temperature Annual Range (P5-P6)	ns	ns
Mean Temperature of Wettest Quarter	ns	ns
Mean Temperature of Driest Quarter	ns	ns
Mean Temperature of Warmest Quarter	ns	ns
Mean Temperature of Coldest Quarter	ns	ns
Annual Precipitation	ns	ns
Precipitation of Wettest Month	ns	-0.18
Precipitation of Driest Month	ns	ns
Precipitation Seasonality	ns	ns
Precipitation of Wettest Quarter	ns	ns
Precipitation of Driest Quarter	ns	ns
Precipitation of Warmest Quarter	ns	ns
Precipitation of Coldest Quarter	ns	ns
Latitude	ns	ns
Longitude	ns	ns

Supplementary Table 3. Primers used in the present study.

Cloning with GreenGate method	
proBSK3pENTR_F	CACCAAACAAACTCTGATGATCAAATCAA
proBSK3pENTR_R	CTAAATAAAACCACGCTCCAAAA
proBSK3_BsaMut_F	GAACGAGTCCACTTGCTTTAACAGG
proBSK3_BsaMut_R	GCAAGTGGACTCGTTCATAGAAC
ggproBSK3_F	AACAGGTCTCAACCTAAACAAACTCTGATGATCAAATCAA
ggproBSK3_R	AACAGGTCTCATGTTCTAAATAAAACCACGCTCCAAAA
ggCvi_BSK3cds(P)_F	AACAGGTCTCAGGCTATGGGAGGTCAATGCTCTA
ggCvi_BSK3cds(P)_R	AACAGGTCTCACTGACTTCACTCGGGGAACTCCAT
ggCol0_BSK3cds(L)_F	AACAGGTCTCAGGCTATGGGAGGTCAATGCTCTA
ggCol0_BSK3cds(L)_R	AACAGGTCTCACTGACTTCACTCGGGGAACTCCAT
For *q*RT-PCR analyses	
UBQ10-*q*PCR-F	CTTCGTCAAGACTTTGACCG
UBQ10-*q*PCR-R	CTTCTTAAGCATAACAGAGACGAG
ACTIN2-*q*PCR-F	GACCAGCTCTTCCATCGAGAA
ACTIN2-*q*PCR-R	CAAACGAGGGCTGGAACAAG
BSK3-*q*PCR-F	TCGAAAGCTTCTGGGTTTACGA
BSK3-*q*PCR-R	AACTCAGGCTAGAGCATTGACC
ASKΘ-*q*PCR-F	GCAGTAGACCTCGTCTCAAGACTC
ASKΘ-*q*PCR-R	GGGTGTGCACAAGCTTCCAATG
BAK1-*q*PCR-F	TGTCCTGACGCTACAAGTTCTGG
BAK1-*q*PCR-R	ACAGGAATATCTCCGGTGAGAGG
BRI1-*q*PCR-F	GTAAACGGCCAACGGATTCACC
BRI1-*q*PCR-R	TTGCGTGCTGTTTCACCCATCC
