# Supplementary material for: Natural variation of BSK3 tunes brassinosteroid signaling to regulate root foraging under low nitrogen
Source: Nat Commun. 2019 May 30;10:2378. doi: 10.1038/s41467-019-10331-9 (PMC6542857; doi:10.1038/s41467-019-10331-9)
Supplement: Supplementary file 3 — Description of Additional Supplementary Files [file 41467_2019_10331_MOESM3_ESM.docx]

**Descriptions of Additional Supplementary Files**

File Name: Supplementary Data 1

Description: List of 200 Arabidopsis accessions screened for primary root length in high vs low nitrogen conditions and presence of L or P at position 319; NA, not available.

File Name: Supplementary Data 2

Description: List of significant marker-trait associations (-log10(P-value)>4) at low nitrogen.

File Name: Supplementary Data 3

Description: Genes located in the QTL associated with primary root length under low nitrogen.

File Name: Supplementary Data 4

Description: List of 56 Arabidopsis accessions assessed for brassinosteroid sensitivity and values for root as well as hypocotyl response to exogenous brassinosteroids.

File Name: Supplementary Data 5

Description: List of 19 climate variables, latitude and longitude of 115 natural accessions used in the study.
