# Supplementary material for: Natural variation of BSK3 tunes brassinosteroid signaling to regulate root foraging under low nitrogen
Source: Nat Commun. 2019 May 30;10:2378. doi: 10.1038/s41467-019-10331-9 (PMC6542857; doi:10.1038/s41467-019-10331-9)
Supplement: Supplementary file 9 — Reporting Summary [file 41467_2019_10331_MOESM9_ESM.pdf]

nature research

Corresponding author(s): Nicolaus von Wirén

Last updated by author(s): Apr 15, 2019

Reporting Summary

Nature Research wishes to improve the reproducibility of the work that we publish. This form provides structure for consistency and transparency in reporting. For further information on Nature Research policies, see Authors & Referees and the Editorial Policy Checklist.

Statistics

For all statistical analyses, confirm that the following items are present in the figure legend, table legend, main text, or Methods section.

n/a	Confirmed	
☐	☒	The exact sample size (*n*) for each experimental group/condition, given as a discrete number and unit of measurement
☐	☒	A statement on whether measurements were taken from distinct samples or whether the same sample was measured repeatedly
☐	☒	The statistical test(s) used AND whether they are one- or two-sided<br>*Only common tests should be described solely by name; describe more complex techniques in the Methods section.*
☒	☐	A description of all covariates tested
☐	☒	A description of any assumptions or corrections, such as tests of normality and adjustment for multiple comparisons
☐	☒	A full description of the statistical parameters including central tendency (e.g. means) or other basic estimates (e.g. regression coefficient) AND variation (e.g. standard deviation) or associated estimates of uncertainty (e.g. confidence intervals)
☐	☒	For null hypothesis testing, the test statistic (e.g. *F*, *t*, *r*) with confidence intervals, effect sizes, degrees of freedom and *P* value noted<br>*Give P values as exact values whenever suitable.*
☒	☐	For Bayesian analysis, information on the choice of priors and Markov chain Monte Carlo settings
☐	☒	For hierarchical and complex designs, identification of the appropriate level for tests and full reporting of outcomes
☐	☒	Estimates of effect sizes (e.g. Cohen's *d*, Pearson's *r*), indicating how they were calculated

Our web collection on statistics for biologists contains articles on many of the points above.

Software and code

Policy information about availability of computer code

Data collection	WinRHIZO Pro 2009 was taken for measuring root length, CFX38TM Real-Time System was used to collect expression data, Elemental Analyzer was used for collecting N concentration
Data analysis	All data analyses were carried in R. Celluar traits were quantified with Zen 2.3. WinRHIZO Pro 2009 was used for measuring root length and ClustalW2.1 for multiple sequence alignment

For manuscripts utilizing custom algorithms or software that are central to the research but not yet described in published literature, software must be made available to editors/reviewers. We strongly encourage code deposition in a community repository (e.g. GitHub). See the Nature Research guidelines for submitting code & software for further information.

Data

Policy information about availability of data

All manuscripts must include a data availability statement. This statement should provide the following information, where applicable:

- Accession codes, unique identifiers, or web links for publicly available datasets
- A list of figures that have associated raw data
- A description of any restrictions on data availability

The authors declare that all data supporting the findings of this study are available within the manuscript and the supplementary files. The source data underlying figures are provided as a Source Data File.

Field-specific reporting

Please select the one below that is the best fit for your research. If you are not sure, read the appropriate sections before making your selection.

☒ Life sciences ☐ Behavioural & social sciences ☐ Ecological, evolutionary & environmental sciences

For a reference copy of the document with all sections, see nature.com/documents/nr-reporting-summary-flat.pdf

Life sciences study design

All studies must disclose on these points even when the disclosure is negative.

Sample size	We chose the sample size based on literatures in the field and our own experience in previous works. The sample sizes used for all experiments provided sufficient resolving power to show statistical significance due to homogeneity of plant individuals.
Data exclusions	Samples were excluded from data analysis when strong deviation in plant growth after transferring from pre-culture to treatments could be clearly associated from unintentional damage caused during seedling transfer. Samples determined as outliers (i.e. lying outside the range of mean ± 3 s.d.) were removed prior to data analysis.
Replication	The number of replication are indicated in the figure legends. All experiments were conducted at least twice.
Randomization	All plants were grown in the same growth medium and in the same growth cabinet. The position of agar plates containing plants in the growth cabinets followed a completely randomized block design.
Blinding	All the experiments were performed without prior knowledge of the final outcome, and therefore blinding was not applied.

Reporting for specific materials, systems and methods

We require information from authors about some types of materials, experimental systems and methods used in many studies. Here, indicate whether each material, system or method listed is relevant to your study. If you are not sure if a list item applies to your research, read the appropriate section before selecting a response.

Materials & experimental systems

n/a	Involved in the study	
☒	☐	Antibodies
☒	☐	Eukaryotic cell lines
☒	☐	Palaeontology
☒	☐	Animals and other organisms
☒	☐	Human research participants
☒	☐	Clinical data

Methods

n/a	Involved in the study	
☒	☐	ChIP-seq
☒	☐	Flow cytometry
☒	☐	MRI-based neuroimaging
